# Surgical Trauma Gradient as an Independent Predictor of Postoperative Pain, Functional Recovery, and Complication Risk After Spine Surgery: A 2 × 2 Invasiveness Model with Psychosocial Interaction

**DOI:** 10.3390/jcm15093189

**Published:** 2026-04-22

**Authors:** Christian Riediger, Mark Ferl, Agnieszka Halm-Pozniak, Christoph H. Lohmann, Maria Schönrogge

**Affiliations:** Orthopaedic University Clinic Magdeburg, Otto-von-Guericke-University Magdeburg, 39120 Magdeburg, Germany; mark.ferl@med.ovgu.de (M.F.); agnieszka.halm-pozniak@med.ovgu.de (A.H.-P.); maria.schoenrogge@med.ovgu.de (M.S.)

**Keywords:** spine surgery, minimally invasive, fusion, decompression, surgical invasiveness, postoperative pain, longitudinal analysis, Type-D personality

## Abstract

**Background/Objective:** Postoperative recovery after spine surgery varies substantially and cannot be fully explained by structural pathology alone. This study evaluates postoperative outcomes using a structured 2 × 2 Surgical Trauma Gradient integrating exposure-related invasiveness (minimally invasive vs. open) and biomechanical strategy (decompression vs. fusion), and examines the modifying role of Type-D personality. **Methods:** This observational cohort study included 200 patients undergoing elective spine surgery. Patients were stratified into four surgical subgroups: minimally invasive decompression, open decompression, minimally invasive fusion, and open fusion. Primary outcomes included pain intensity (Visual Analog Scale), functional disability (Oswestry Disability Index), patient satisfaction (Patient Satisfaction Index), and postoperative complications at 12-month follow-up. Surgical invasiveness was modeled both categorically and as an ordinal gradient. Multivariable regression, logistic regression, interaction analysis, and longitudinal mixed-effects models were applied. **Results:** Postoperative outcomes demonstrated a consistent gradient across increasing surgical burden. In multivariable models, higher surgical invasiveness independently predicted greater residual pain (β = 0.69; 95% CI 0.55–0.82; *p* < 0.001) and higher functional disability (β = 6.20; 95% CI 5.10–7.30; *p* < 0.001). Increasing invasiveness was also associated with lower patient satisfaction (β = −0.38; 95% CI −0.47 to −0.29; *p* < 0.001) and higher complication risk (OR = 1.64; 95% CI 1.12–2.41; *p* = 0.01). Type-D personality independently predicted worse postoperative pain (β = 0.41; *p* = 0.008) and significantly modified the association between surgical burden and pain (interaction β = 0.22; *p* = 0.012). **Conclusions:** Postoperative outcomes follow a structured Surgical Trauma Gradient influenced by both surgical burden and psychosocial vulnerability, particularly Type-D personality. Integrating these dimensions may improve perioperative risk stratification and support individualized treatment strategies.

## 1. Introduction

Low back pain (LBP) and degenerative spine disorders remain among the most prevalent and disabling conditions worldwide. According to the Global Burden of Disease Study 2019, low back pain continues to rank as one of the leading causes of years lived with disability across all age groups and geographic regions [1]. The lifetime prevalence in Western populations approaches 70–80%, and a substantial proportion of affected individuals develop persistent or recurrent symptoms requiring specialist evaluation [1,2]. Importantly, structural abnormalities detected on imaging frequently correlate only weakly with pain intensity or functional impairment, underscoring the multifactorial nature of spine-related pain syndromes [2].

The biopsychosocial model has become central to understanding chronic musculoskeletal pain. Beyond nociceptive input and structural degeneration, affective, cognitive, and behavioral factors critically shape symptom perception, coping strategies, and recovery trajectories [3,4]. Fear-avoidance beliefs, catastrophizing, and maladaptive coping have been shown to contribute to pain chronification and functional decline [3,4]. In this context, stable personality traits may act as vulnerability factors that predispose individuals to heightened stress reactivity and persistent symptom amplification.

Among such traits, Type-D personality, defined by the combination of high negative affectivity (NA) and high social inhibition (SI), has emerged as a clinically relevant psychosocial construct [5,6,7]. Initially described in cardiovascular populations [6,7,8,9,10], Type-D personality has subsequently been associated with adverse outcomes in various chronic conditions, including musculoskeletal disorders and chronic pain syndromes [5,11,12,13,14,15,16,17,18]. Individuals with Type-D personality tend to experience persistent emotional distress, impaired stress coping, and reduced social support utilization [8,17,19]. In chronic pain populations, Type-D personality has been linked to greater pain intensity, higher disability, and poorer quality of life [5,11,12,13,14,18].

Our recent cross-sectional study in a university spine outpatient clinic demonstrated that approximately one-third of patients met criteria for Type-D personality and reported significantly higher pain intensity, functional disability, anxiety, and depressive symptoms compared with non-Type-D patients [18]. Moreover, in a surgical spine cohort, we previously showed that Type-D personality independently predicted postoperative pain and functional outcomes following spine surgery [20]. These findings suggest that psychological vulnerability may exert clinically meaningful effects both prior to and after surgical intervention.

However, while the psychosocial dimension has increasingly been recognized, the surgical dimension itself is often conceptualized in overly simplistic terms. Spine surgery is commonly categorized dichotomously as minimally invasive versus open or decompression versus fusion. Such binary classifications, however, do not adequately reflect the multidimensional burden of surgical trauma. Surgical exposure influences paraspinal muscle injury, blood loss, inflammatory activation, and postoperative pain generation, whereas biomechanical strategy (decompression versus stabilization/fusion) fundamentally alters segmental motion and load distribution. These axes likely exert additive, and potentially interactive, effects on postoperative recovery.

Persistent pain after spinal surgery remains a significant clinical problem. A recent systematic review and meta-analysis reported a substantial prevalence of chronic pain following spinal procedures [21]. Psychological factors, including personality traits and preoperative anxiety, have been shown to influence postoperative trajectories [22,23,24]. Nevertheless, few studies have integrated psychosocial vulnerability with a structured model of surgical invasiveness.

Beyond behavioral mechanisms, biological pathways may further explain the interaction between psychological vulnerability and surgical burden. Type-D personality has been associated with autonomic dysregulation, increased sympathetic activation, reduced vagal tone, and elevated inflammatory markers [9,25]. The cholinergic anti-inflammatory pathway, mediated by vagal efferents, plays a key role in modulating cytokine production and systemic inflammatory responses [26,27]. Dysregulation of this pathway has been implicated in chronic inflammatory states and pain sensitization. Experimental and clinical studies suggest that vagus nerve stimulation can attenuate inflammatory signaling and modulate pain perception [28,29,30,31,32]. Although these mechanisms were not directly assessed in the present study, they provide a plausible biological framework linking personality traits, surgical stress response, and chronic pain development.

Taken together, existing literature suggests that both psychosocial vulnerability and surgical burden independently influence outcomes after spine surgery. However, an integrative framework capturing both dimensions is lacking.

Therefore, the present study introduces a 2 × 2 Surgical Trauma Gradient model, integrating:

1. Exposure-related burden (minimally invasive vs. open surgery)
2. Biomechanical burden (decompression vs. fusion/stabilization)

This framework generates four clinically meaningful subgroups:

- Minimally Invasive Decompression (MIS-D)
- Open Decompression (O-D)
- Minimally Invasive Fusion (MIS-F)
- Open Fusion (O-F)

By modeling surgical invasiveness as an ordinal gradient rather than a binary variable, we aim to test whether postoperative pain, disability, satisfaction, and complication risk follow a graded relationship across increasing surgical burden. In addition, building upon our prior findings regarding Type-D personality in spine populations, we explore whether psychological vulnerability interacts with surgical burden to further influence recovery trajectories.

The primary objective of this study was to determine whether postoperative outcomes after spine surgery follow a graded relationship across a structured 2 × 2 Surgical Trauma Gradient integrating exposure-related invasiveness and biomechanical strategy.

Secondary objectives were to evaluate whether Type-D personality independently predicts postoperative outcomes and whether psychosocial vulnerability modifies the association between surgical burden and postoperative recovery.

## 2. Materials and Methods

### 2.1. Study Design and Conceptual Framework

This observational cohort study applies a structured 2 × 2 Surgical Trauma Gradient framework to evaluate postoperative outcomes following elective spine surgery. The model integrates two independent dimensions of surgical burden: (i) exposure-related invasiveness (minimally invasive vs. open approach) and (ii) biomechanical strategy (decompression vs. fusion/stabilization).

This design defines four predefined subgroups: minimally invasive decompression (MIS-D), open decompression (O-D), minimally invasive fusion (MIS-F), and open fusion (O-F). In addition to categorical subgroup comparisons, an ordinal InvasivenessScore (1–4) was assigned to model a graded relationship (1 = MIS-D; 2 = O-D; 3 = MIS-F; 4 = O-F), enabling the assessment of dose–response relationships of surgical trauma.

The four-group structure was chosen to capture the two most clinically relevant dimensions of surgical burden, tissue exposure and biomechanical alteration, while maintaining interpretability and statistical robustness. Furthermore, psychosocial vulnerability, specifically Type-D personality, was incorporated as an independent predictor and interaction variable to evaluate its modifying effect on postoperative outcomes.

### 2.2. Participants

This prospective cohort study was conducted at the Department of Orthopedics, University Hospital Magdeburg, between March 2022 and December 2024. Adult patients aged 18–75 years scheduled for elective spine surgery for degenerative lumbar or cervical spine disorders were eligible. Surgical indications included lumbar spinal stenosis, lumbar or cervical disk herniation, degenerative spondylolisthesis, and segmental instability. All procedures were performed at a tertiary academic spine center.

To ensure clinical representativeness, patients were included irrespective of pain duration or prior conservative treatment. Pain duration was systematically assessed at baseline using a standardized patient questionnaire and recorded in months. It was pre-specified as a clinically relevant variable and included as a covariate in multivariable analyses and as a stratification variable in exploratory subgroup analyses.

Exclusion criteria comprised previous spine surgery, acute traumatic spinal injury, spinal tumors or infections, severe cognitive impairment precluding reliable questionnaire completion, insufficient German language proficiency, and major psychiatric disorders requiring acute treatment. Major psychiatric disorders were defined as previously diagnosed psychotic disorders, bipolar disorder, or severe major depressive episodes requiring inpatient or acute psychiatric care. Patients with relevant comorbidities that could substantially influence pain perception or functional outcomes were also excluded, including advanced heart failure (New York Heart Association (NYHA) class III–IV), insulin-dependent diabetes with neuropathy, chronic kidney disease stage ≥ 4, neurodegenerative disorders (e.g., multiple sclerosis or Parkinson’s disease), and prior cerebrovascular events with persistent neurological deficits.

All participants received written and oral information about the study and provided written informed consent prior to enrollment.

### 2.3. Surgical Classification

Surgical procedures were categorized based on operative reports. Operative characteristics such as number of operated levels, operative time, and estimated blood loss were recorded but were not incorporated into the gradient model in order to preserve conceptual clarity.

Procedures were classified as follows:

(1) Minimally Invasive Decompression (MIS-D): Tubular or endoscopic microdiscectomy or decompression with minimal paraspinal muscle detachment and no instrumentation.

(2) Open Decompression (O-D): Standard midline approach with bilateral muscle detachment and decompression without instrumentation.

(3) Minimally Invasive Fusion (MIS-F): Percutaneous pedicle screw fixation with limited exposure and interbody fusion (e.g., transforaminal lumbar interbody fusion (TLIF)).

(4) Open Fusion (O-F): Conventional open posterior instrumentation with or without interbody fusion using a midline approach.

This classification reflects increasing degrees of paraspinal muscle injury, operative exposure, biomechanical alteration, and inflammatory burden.

Operative characteristics such as operative time, estimated blood loss, and number of operated levels were recorded but deliberately not incorporated into the gradient model in order to preserve conceptual parsimony and maintain a clinically interpretable framework. In cases of intraoperative conversion from minimally invasive to open surgery, classification was assigned according to the final surgical technique. Intraoperative adverse events were recorded as complications but did not alter subgroup classification.

### 2.4. Psychosocial Assessment

Type-D personality was assessed using the validated 14-item Type-D Scale (DS14), which measures the two core dimensions negative affectivity (NA) and social inhibition (SI) [6]. Each subscale consists of seven items rated on a 5-point Likert scale, yielding subscale scores ranging from 0 to 28. In accordance with established criteria, patients scoring ≥10 on both NA and SI were classified as having a Type-D personality.

The DS14 has demonstrated robust internal consistency and construct validity across cardiovascular and musculoskeletal populations [5,6,9,10,11,12,13,14]. In the validated German version, internal consistency has been reported as Cronbach’s α = 0.87 for negative affectivity and α = 0.86 for social inhibition [33].

### 2.5. Clinical Outcome Measures

Clinical outcomes were assessed preoperatively and at 3, 6, and 12 months postoperatively using validated instruments commonly applied in spine research.

#### 2.5.1. Pain Intensity

Pain intensity was measured using a Visual Analog Scale (VAS, 0–10), where 0 represents “no pain” and 10 represents “worst imaginable pain” [34].

#### 2.5.2. Functional Disability

Functional disability was assessed using the Oswestry Disability Index (ODI, 0–100), which quantifies spine-related limitations in activities of daily living, with higher scores indicating greater disability [35].

#### 2.5.3. Patient Satisfaction

Patient-reported satisfaction was assessed using the Patient Satisfaction Index (PSI, 1–5), a composite patient-reported outcome measure assessing overall satisfaction, perceived improvement, and willingness to undergo the procedure again, with higher values reflecting greater satisfaction [36].

#### 2.5.4. Complications

Defined as any postoperative adverse event requiring medical or surgical intervention within 12 months.

All instruments have demonstrated strong reliability, validity, and sensitivity to clinical change in orthopedic and spine-surgery populations. All evaluations were performed by trained research assistants blinded to surgical allocation.

#### 2.5.5. Additional Covariates

Baseline covariates included age, sex, body mass index (BMI), pain duration (months), prior spine surgery, and comorbid depression diagnosis. Pain duration was treated as a continuous variable and included in all multivariable models.

### 2.6. Statistical Analysis

Statistical analyses were conducted using advanced multivariate modeling strategies. A two-sided *p*-value < 0.05 was considered statistically significant.

#### 2.6.1. Descriptive Analysis

Continuous variables are presented as means ± standard deviations. Categorical variables are presented as frequencies and percentages. Normality was assessed using Shapiro–Wilk testing in combination with visual inspection of histograms and Q–Q plots.

#### 2.6.2. Group Comparisons

Differences between the four surgical subgroups were analyzed using one-way ANOVA for VAS, ODI, and PSI. Post hoc comparisons were performed using Tukey correction. Effect sizes were calculated using Cohen’s d.

#### 2.6.3. Multivariable Linear Regression

Multivariable linear regression models were constructed to evaluate independent associations between surgical burden and postoperative outcomes:

- VAS_12_m~InvasivenessScore + Age + Sex + BMI + PainDuration + TypeD
- ODI_12_m~InvasivenessScore + Age + Sex + BMI + PainDuration + TypeD
- PSI_12_m~InvasivenessScore + Age + Sex + BMI + PainDuration + TypeD

Results are reported as unstandardized regression coefficients (β) with 95% confidence intervals. Model fit was assessed using adjusted R^2^.

#### 2.6.4. Logistic Regression for Complications

Complication risk was analyzed using multivariable logistic regression:

- Complication~InvasivenessScore + Age + Sex + BMI + TypeD

Results are reported as odds ratios (OR) with 95% confidence intervals.

#### 2.6.5. Interaction Analysis (Type-D × Invasiveness)

To assess effect modification by psychosocial vulnerability, an interaction term between Type-D personality and surgical invasiveness was introduced:

- VAS_12_m~InvasivenessScore × TypeD + covariates

This model evaluates whether the slope of surgical burden differs between Type-D and non-Type-D patients.

#### 2.6.6. Longitudinal Mixed-Effects Modeling

Recovery trajectories were analyzed using linear mixed-effects models:

- VAS~InvasivenessScore × Month + TypeD + covariates

Random intercept models with unstructured covariance were applied to account for intra-individual correlation. Month was treated as a continuous variable. This approach allows adjustment for intra-individual correlation, inclusion of incomplete follow-up data, and estimation of differences in recovery dynamics across subgroups.

#### 2.6.7. Sensitivity Analyses

Sensitivity analyses included separate modeling of exposure and biomechanical strategy, exclusion of patients with prior spine surgery, and stratification by pain duration quartiles. Model assumptions were evaluated using residual diagnostics and variance inflation factors, with no evidence of relevant violations.

Model assumptions were assessed using residual diagnostics, including visual inspection of residual plots and tests for normality and homoscedasticity. Multicollinearity was evaluated using variance inflation factors (VIF), with no evidence of significant collinearity observed. Model fit was assessed using adjusted R^2^ for linear models and goodness-of-fit metrics for logistic regression. The analytical framework was designed to illustrate the gradient-based modeling approach rather than to establish definitive causal estimates. Accordingly, findings should be interpreted as hypothesis-generating and supportive of the proposed conceptual framework.

#### 2.6.8. Sample Size Considerations

Given the observational nature of this cohort study, no formal a priori sample size calculation was performed. The study included all consecutive eligible patients treated during the predefined study period.

To provide context for statistical adequacy, the available sample size of 200 patients allowed detection of small-to-moderate effect sizes (Cohen’s d ≈ 0.35) with a two-sided α level of 0.05 and a statistical power of 80% in continuous outcomes such as pain intensity (VAS) and functional disability (ODI). The effect sizes observed in the present analyses were within or above this range, supporting the robustness of the findings. The sample size was also sufficient to support multivariable regression and interaction analyses with an appropriate ratio of predictors to observations.

Nevertheless, the results should be interpreted within the context of an observational and partly hypothesis-generating design.

#### 2.6.9. Ethical Considerations

The study was conducted in accordance with the Declaration of Helsinki and approved by the Ethics Committee of the Otto-von-Guericke University Magdeburg (protocol codes 30/22, approval date: 28 February 2022 and 172/15, approval date: 14 December 2015). All participants provided written informed consent. This study was conducted and reported in accordance with the STROBE (Strengthening the Reporting of Observational Studies in Epidemiology) guidelines for cohort studies.

## 3. Results

### 3.1. Patient Characteristics and Surgical Distribution

A total of 200 patients undergoing elective spine surgery were included and stratified according to the predefined 2 × 2 Surgical Trauma Gradient into four subgroups: minimally invasive decompression (MIS-D; *n* = 60), open decompression (O-D; *n* = 50), minimally invasive fusion (MIS-F; *n* = 45), and open fusion (O-F; *n* = 45).

Baseline demographic characteristics were comparable across groups with respect to age, body mass index, and Type-D personality distribution (Table 1). Preoperative pain intensity and disability showed a gradual increase across the gradient, consistent with increasing disease severity. There were no clinically relevant between-group differences in pain duration or comorbid depression diagnosis, supporting comparability of the study population.

### 3.2. Twelve-Month Outcomes Across the Surgical Trauma Gradient

#### 3.2.1. Pain Intensity (VAS)—Twelve-Month Outcomes

At 12 months postoperatively, pain intensity demonstrated a clear monotonic increase across the Surgical Trauma Gradient (Table 2). Patients undergoing MIS-D reported the lowest residual pain (VAS 2.12), followed by O-D (2.60), MIS-F (3.26), and O-F (4.13). The overall group difference was statistically significant (ANOVA, *p* < 0.001).

**Figure 1:**
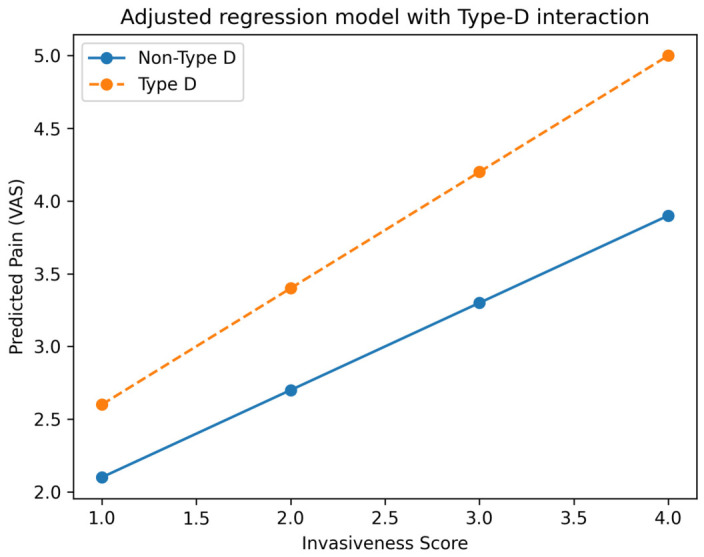
Adjusted regression model demonstrating the interaction between surgical invasiveness and Type-D personality on postoperative pain (VAS). Predicted pain values are shown across the InvasivenessScore (1–4), derived from multivariable regression analysis. Patients with Type-D personality exhibit a steeper increase in pain across the Surgical Trauma Gradient, consistent with a significant interaction effect (β = 0.22; *p* = 0.012). This finding indicates that psychosocial vulnerability amplifies the impact of surgical burden on postoperative pain.

As illustrated in Figure 1, adjusted model predictions confirmed a graded increase in pain across the InvasivenessScore, with a steeper slope observed in Type-D patients, indicating a statistically significant effect modification by psychosocial vulnerability. Post hoc comparisons demonstrated significantly higher pain levels in open versus minimally invasive procedures and in fusion versus decompression procedures, with the highest residual pain observed in the open fusion group. Effect sizes were in the moderate-to-large range, indicating clinically meaningful differences.

#### 3.2.2. Functional Disability (ODI)—Twelve-Month Outcomes

Functional disability followed a similar gradient pattern (Table 2). ODI scores increased from 12.36 in the MIS-D group to 29.53 in the O-F group (*p* < 0.001). The magnitude of difference between the lowest and highest burden groups exceeded established minimal clinically important differences, supporting both statistical and clinical relevance of the observed gradient.

#### 3.2.3. Patient Satisfaction (PSI)—Twelve-Month Outcomes

**Figure 2:**
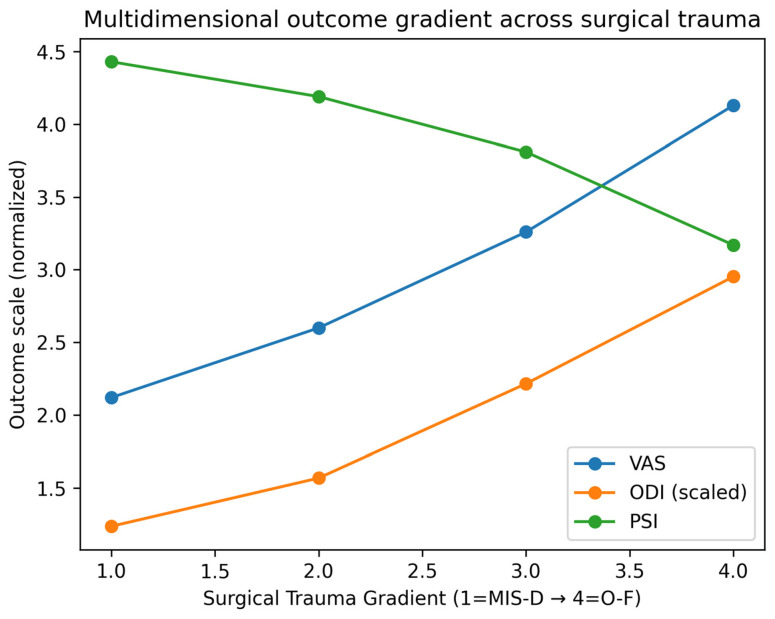
Multidimensional outcome gradient across the Surgical Trauma Gradient (MIS-D → O-F). Mean values of postoperative pain intensity (VAS), functional disability (ODI), and patient satisfaction (PSI) at 12 months are displayed across increasing levels of surgical burden. ODI values are scaled for visualization purposes. A consistent monotonic trend is observed, with increasing surgical invasiveness associated with higher residual pain and disability and lower patient satisfaction. This figure illustrates the multidimensional nature of postoperative outcome deterioration along the Surgical Trauma Gradient.

Patient satisfaction decreased proportionally with increasing surgical burden (Table 2). PSI values declined from 4.43 in MIS-D to 3.17 in O-F (*p* < 0.001), demonstrating an inverse gradient that mirrors the patterns observed for pain and disability. This finding underscores the consistency of the Surgical Trauma Gradient across multiple outcome domains (Figure 2).

#### 3.2.4. Complication Rates

**Figure 3:**
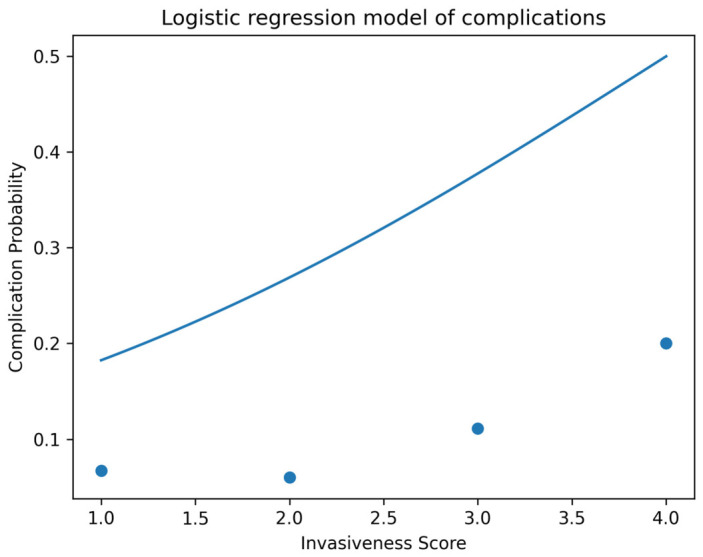
Logistic regression model of postoperative complication risk across the Surgical Trauma Gradient. The continuous curve represents the predicted probability of complications derived from the multivariable logistic regression model, while data points indicate observed group-level complication rates. Increasing surgical invasiveness is associated with a higher probability of complications (OR = 1.64 per level increase; *p* = 0.01), demonstrating a dose–response relationship between surgical burden and adverse events.

Postoperative complication rates increased stepwise across the Surgical Trauma Gradient (Table 2), ranging from 6.7% in MIS-D to 20.0% in O-F. Reported complications included wound-related complications, postoperative hematoma requiring intervention, neurological symptoms, and medical adverse events. Multivariable logistic regression confirmed that each incremental increase in InvasivenessScore was associated with higher odds of complications (OR = 1.64; 95% CI 1.12–2.41; *p* = 0.01), as visualized in Figure 3. This association remained robust after adjustment for demographic and clinical covariates.

### 3.3. Multivariable Linear Regression Analyses

#### 3.3.1. Pain Intensity (VAS)

In multivariable linear regression, InvasivenessScore remained a strong independent predictor of 12-month pain (β = 0.69; 95% CI 0.55–0.82; *p* < 0.001; Table 3). Each incremental increase in surgical burden corresponded to a clinically relevant rise in residual pain. Type-D personality was independently associated with higher pain levels (β = 0.41; *p* = 0.008), confirming its role as a psychosocial risk factor. The model explained a substantial proportion of variance, indicating robust predictive capacity.

#### 3.3.2. Functional Disability (ODI)

InvasivenessScore independently predicted postoperative disability (β = 6.20; 95% CI 5.10–7.30; *p* < 0.001; Table 3). Type-D personality also contributed independently to higher ODI scores (β = 3.10; *p* = 0.002). These findings suggest additive effects of surgical burden and psychosocial vulnerability on functional outcomes.

#### 3.3.3. Patient Satisfaction (PSI)

Higher surgical invasiveness was independently associated with reduced patient satisfaction (β = −0.38; 95% CI −0.47 to −0.29; *p* < 0.001; Table 3). Although Type-D personality showed a negative association with satisfaction, the effect of surgical burden remained the dominant predictor.

### 3.4. Interaction Analysis: Type-D Personality × Invasiveness

Interaction analysis revealed a statistically significant interaction between Type-D personality and InvasivenessScore for postoperative pain (β = 0.22; 95% CI 0.05–0.39; *p* = 0.012; Table 3). This indicates that the increase in pain across the Surgical Trauma Gradient is more pronounced in Type-D patients. In other words, psychologically vulnerable individuals exhibit a steeper slope of worsening pain with increasing surgical burden (Figure 1). This confirms a statistically significant effect modification rather than a descriptive trend. Predicted marginal effects indicated that Type-D patients exhibited approximately 0.6–0.8 higher VAS points at the highest invasiveness level compared with non-Type-D patients, illustrating the clinical relevance of the interaction effect (Supplementary Table S2 and Supplementary Figure S1).

The interaction analysis was pre-specified and interpreted with caution given the observational study design. Overall, these findings support a moderating role of psychosocial vulnerability in postoperative recovery.

### 3.5. Longitudinal Recovery Trajectories

**Figure 4:**
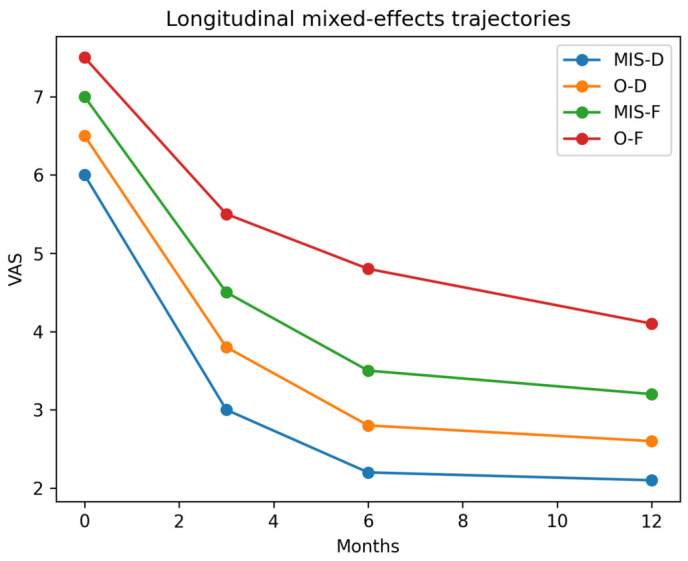
Longitudinal trajectories of postoperative pain (VAS) across surgical subgroups based on mixed-effects modeling. Pain levels are shown at baseline, 3, 6, and 12 months postoperatively. Distinct recovery patterns are observed, with minimally invasive decompression (MIS-D) demonstrating the fastest improvement, while open fusion (O-F) shows persistently elevated pain levels. The significant Time × Invasiveness interaction indicates that recovery dynamics differ systematically across the Surgical Trauma Gradient.

Linear mixed-effects models demonstrated a significant reduction in pain over time across all subgroups (*p* < 0.001), reflecting postoperative recovery (Figure 4). However, higher InvasivenessScore was associated with persistently elevated pain levels throughout follow-up.

A significant Time × Invasiveness interaction indicated differential recovery trajectories. Patients undergoing minimally invasive decompression showed the fastest improvement, whereas those undergoing open fusion exhibited slower and attenuated recovery. These results suggest that surgical burden influences both the magnitude and trajectory of postoperative pain.

### 3.6. Results of Sensitivity Analyses

Sensitivity analyses were performed by modeling exposure (minimally invasive vs. open) and biomechanical strategy (decompression vs. fusion) as separate predictors. Both dimensions remained independently associated with postoperative pain and disability, supporting the conceptual validity of the 2 × 2 framework.

Exclusion of patients with prior spine surgery and stratification by pain duration quartiles did not materially alter the direction or magnitude of the main findings.

Detailed regression coefficients and stratified estimates are provided in Supplementary Tables S1 and S2, and graphical visualization is shown in Supplementary Figure S1.

Stratified analyses by pain duration suggested that longer preoperative symptom duration was associated with increased postoperative disability, particularly in higher-burden procedures. These findings should be interpreted as exploratory.

### 3.7. Summary of Key Findings

Across all outcome domains, a consistent gradient was observed with increasing surgical burden. At 12 months, residual pain increased from 2.12 in minimally invasive decompression to 4.13 in open fusion, while disability increased from 12.36 to 29.53 and satisfaction decreased from 4.43 to 3.17.

In multivariable models, InvasivenessScore independently predicted pain (β = 0.69; *p* < 0.001), disability (β = 6.20; *p* < 0.001), lower satisfaction (β = −0.38; *p* < 0.001), and complication risk (OR = 1.64; *p* = 0.01).

Type-D personality independently predicted worse pain (β = 0.41; *p* = 0.008) and significantly amplified the effect of surgical burden on postoperative pain (interaction β = 0.22; *p* = 0.012).

Higher-burden procedures were additionally associated with slower recovery trajectories, supporting a dose–response relationship between surgical invasiveness and postoperative outcome. Collectively, these findings support the validity of a gradient-based model integrating surgical and psychosocial determinants of postoperative outcome (Table 2, Table 3 and Table 4; Figure 1, Figure 2, Figure 3 and Figure 4).

## 4. Discussion

This study demonstrates that postoperative recovery after spine surgery follows a consistent and clinically meaningful Surgical Trauma Gradient. By integrating two fundamental dimensions of surgical burden, exposure-related tissue trauma and biomechanical strategy, our 2 × 2 framework provides a structured and clinically interpretable model for understanding postoperative variability. Importantly, this approach moves beyond traditional binary categorizations such as minimally invasive versus open surgery or decompression versus fusion and instead conceptualizes surgical trauma as a graded determinant of postoperative outcome.

The main finding of the present study is that increasing surgical burden was associated with progressively worse pain, greater disability, lower patient satisfaction, and a higher probability of postoperative complications. This gradient remained robust across descriptive analyses, multivariable models, and longitudinal recovery trajectories, suggesting that surgical burden not only affects final symptom levels but also shapes the speed and pattern of postoperative recovery. In this context, the proposed Surgical Trauma Gradient should be interpreted primarily as a conceptual modeling framework rather than a definitive classification system. While the four-group structure captures clinically meaningful aspects of operative exposure and biomechanical alteration, refinement in future multicenter cohorts will be necessary.

These findings extend prior work on postoperative outcome variability by suggesting that the magnitude of surgical trauma constitutes a measurable burden that may be modeled more precisely than with conventional dichotomous classifications. In our framework, decompression procedures were consistently associated with more favorable outcomes than fusion procedures, while minimally invasive approaches showed advantages over open exposure. Together, these findings support the premise that operative exposure and biomechanical intervention contribute additively to postoperative burden. At the same time, it must be acknowledged that fusion procedures are often performed in patients with more advanced structural pathology, which may partly contribute to the observed differences. Although the multivariable models accounted for key demographic and clinical covariates, residual confounding related to indication severity cannot be completely excluded. Although residual confounding related to surgical indication and disease severity cannot be fully excluded in an observational design, the persistence of the graded association after multivariable adjustment and across longitudinal analyses suggests that surgical burden contributes independently to outcome variability. In other words, the observed gradient is unlikely to be explained solely by baseline case complexity.

A plausible mechanistic explanation is that larger surgical exposure and stabilization procedures impose a greater nociceptive and inflammatory load, thereby facilitating the transition from acute postoperative pain to persistent pain. Higher complication rates observed in open fusion procedures likely reflect both increased surgical burden and underlying disease severity, although residual confounding cannot be fully excluded. Chronic postsurgical pain is increasingly understood as a multifactorial process in which perioperative tissue damage, inflammatory activation, and central sensitization converge. This interpretation is consistent with broader concepts of pain chronification in musculoskeletal disorders, where sustained nociceptive input interacts with cognitive and affective risk factors [3,4]. Fusion procedures generally involve greater paraspinal muscle detachment, wider tissue disruption, and prolonged biomechanical alteration, which likely increase both the intensity and duration of nociceptive signaling. In contrast, minimally invasive procedures may reduce muscle injury, preserve tissue perfusion, and attenuate local inflammatory cascades. The slower recovery slopes observed in higher-burden procedures support the interpretation that increased surgical trauma delays not only symptom resolution but also functional restoration.

Beyond mechanical and nociceptive explanations, inflammatory mechanisms likely represent a key biological mediator linking surgical burden to recovery outcomes. Surgical trauma induces a coordinated stress response involving cytokine release and immune activation. Persistent inflammatory signaling can contribute to peripheral sensitization, neuroimmune activation, and central nervous system changes associated with chronic pain. The relevance of this pathway is supported by prior evidence linking Type-D personality with increased vulnerability to depressive symptoms and psychosocial stress, potentially mediated through inflammation and endothelial dysfunction [25]. Although inflammatory biomarkers were not assessed in the present study, the convergence of these findings suggests that the inflammatory axis may play a central role in the gradient relationship observed here.

An additional and particularly compelling mechanistic framework is provided by the autonomic nervous system. The cholinergic anti-inflammatory pathway, mediated by vagal efferents, has been shown to modulate cytokine release and attenuate systemic inflammation [26,27]. Reduced vagal tone and increased sympathetic activation have been associated with stress-related vulnerability states and may contribute to exaggerated inflammatory responses and increased pain sensitivity. Type-D personality is characterized by persistent negative affectivity and social inhibition, traits that are frequently linked to autonomic imbalance, chronic stress activation, and maladaptive coping [8,9]. In this context, the observed interaction between Type-D personality and surgical burden appears biologically plausible: individuals with impaired stress regulation may be less capable of buffering the inflammatory and nociceptive consequences of high-burden procedures. This may contribute to prolonged pain trajectories, delayed recovery, and possibly increased complication susceptibility.

The translational implications of this interpretation are notable. Recent studies have suggested that non-invasive vagus nerve stimulation may exert clinically meaningful effects on chronic pain and inflammatory regulation [28,29,30,31,32]. Although the present study did not directly investigate neuromodulation, the current findings support the hypothesis that patients undergoing high-burden surgery, particularly those with elevated psychosocial vulnerability, may represent a relevant target population for future perioperative interventions aimed at restoring autonomic balance and attenuating neuroinflammatory sensitization.

A central contribution of the present study is the integration of Type-D personality into the Surgical Trauma Gradient model. Type-D personality has been extensively validated across chronic disease populations and has repeatedly been associated with worse pain, disability, and quality of life [5,6,9,10,11,12,13,14,15,16,17]. In musculoskeletal and orthopedic settings, it has been linked to increased symptom burden, dysfunctional outcomes, and reduced postoperative recovery [13,20]. The current findings extend this evidence by showing that Type-D personality is not only an independent predictor of worse postoperative pain and disability, but also a modifier of the relationship between surgical burden and recovery. The significant interaction effect indicates that the slope of worsening pain across the Surgical Trauma Gradient is steeper in psychologically vulnerable individuals.

Several psychosocial mechanisms may explain this effect. Type-D individuals often exhibit heightened threat appraisal, maladaptive coping, reduced coping flexibility, and increased avoidance behavior, all of which are central components of fear-avoidance models of disability [4]. Reduced perceived social support may further impair engagement with rehabilitation and postoperative self-management [17]. Importantly, Type-D personality represents a stable personality construct rather than a transient mood state such as depression or anxiety [6,9]. Although conceptual overlap between negative affectivity and depressive symptoms exists, the DS14 captures enduring vulnerability traits that influence stress perception and behavioral responses over time. This distinction strengthens the interpretation of Type-D personality as a clinically meaningful vulnerability factor rather than simply a proxy for current psychological distress.

The findings also have direct clinical implications. First, the Surgical Trauma Gradient offers a simple and interpretable framework for preoperative counseling and expectation management. Patients and clinicians may benefit from a more nuanced understanding of how operative exposure and biomechanical intervention jointly influence recovery. Second, when anatomical and biomechanical conditions permit, minimizing surgical burden may improve postoperative outcomes, especially in psychologically vulnerable patients. Third, psychosocial screening with brief instruments such as the DS14 may help identify patients at elevated risk for persistent postoperative pain and delayed recovery. Such patients may benefit from perioperative prehabilitation strategies incorporating psychological support, coping-oriented interventions, and enhanced multidisciplinary follow-up. Finally, the current model may support the development of individualized perioperative care pathways in which surgical decision-making and postoperative management are informed by both structural and psychosocial risk factors. Routine preoperative screening using the DS14 may help identify patients at risk. In patients with Type-D personality, targeted prehabilitation strategies, including psychological support and expectation management, may be particularly beneficial. Patients undergoing high-burden procedures—especially those with psychosocial vulnerability—may benefit from structured perioperative support and enhanced follow-up.

This study has several limitations. First, the proposed subgroup framework, while clinically meaningful, remains simplified and may contain heterogeneity within categories, particularly among fusion procedures. Second, objective intraoperative burden measures such as operative time, blood loss, or number of operated levels were not incorporated into the gradient model and should be included in future refinements. Third, mechanistic biomarkers such as cytokines or heart rate variability (HRV) were not assessed; consequently, the proposed inflammatory and autonomic interpretations remain hypothesis-generating. Fourth, although 12-month follow-up captures most postoperative recovery dynamics, longer-term outcomes may provide additional insight into chronic pain trajectories and implant-related complications. Fifth, causal inference cannot be established due to the observational design. Unmeasured confounding related to surgical indication remains a relevant limitation. As this study was conducted in a single tertiary academic center, external validity may be influenced by institutional factors. Finally, as with all observational modeling studies, residual confounding cannot be fully excluded.

Future studies should extend the Surgical Trauma Gradient framework by incorporating objective measures of disease severity, neurological status, quality of life, and functional recovery, as well as biological markers, to further refine risk stratification and mechanistic understanding. In addition, interventional studies should assess whether psychosocial prehabilitation, risk-adapted surgical strategy, or autonomic modulation can improve outcomes in high-risk subgroups. Taken together, the present findings support the conceptual value of integrating surgical burden and psychosocial vulnerability into a unified and clinically meaningful framework for postoperative outcome modeling in spine surgery.

To our knowledge, this is the first study to integrate surgical invasiveness and psychosocial vulnerability into a unified gradient-based model for postoperative outcome prediction in spine surgery. The consistency of findings across multivariable models, interaction analysis, and graphical visualization (Figure 1, Figure 2, Figure 3 and Figure 4) supports the robustness of the proposed framework.

## 5. Conclusions

Postoperative outcomes after spine surgery follow a consistent gradient of surgical burden, reflecting the combined effects of exposure-related tissue injury and biomechanical intervention. Increasing surgical burden was independently associated with higher residual pain, greater functional disability, lower patient satisfaction, and increased complication risk.

Type-D personality emerged as an independent psychosocial risk factor and significantly modified the association between surgical burden and postoperative pain.

The proposed 2 × 2 Surgical Trauma Gradient model provides a clinically applicable framework that may support surgical decision-making, preoperative risk stratification, patient counseling, and targeted postoperative follow-up. Importantly, the proposed classification can be readily implemented in current clinical practice without additional resources, supporting surgical planning, psychosocial risk stratification, patient counseling, and individualized postoperative care pathways.

Future studies should validate this framework in multicenter cohorts, incorporate objective operative metrics, and evaluate targeted perioperative interventions.

## Figures and Tables

**Table 1:** Baseline characteristics of the study population.

Variable	MIS-D	O-D	MIS-F	O-F
N	60	50	45	45
Age (years)	51.5	52.3	51.5	50.6
BMI	27.6	26.9	26.8	27.5
Type-D personality (%)	33%	32%	24%	20%
Baseline VAS	6.5	6.7	7.1	7.4
Baseline ODI	32.4	36.8	44.2	52.6

**Table 2:** Twelve-month clinical outcomes across surgical subgroups.

Outcome	MIS-D	O-D	MIS-F	O-F
VAS (12 months)	2.12	2.60	3.26	4.13
ODI (12 months)	12.36	15.68	22.16	29.53
PSI (12 months)	4.43	4.19	3.81	3.17
Complication rate	6.7%	6.0%	11.1%	20.0%

**Table 3:** Multivariable linear regression models for postoperative outcomes.

Outcome	Predictor	β	95% CI	*p*
VAS	InvasivenessScore	0.69	0.55–0.82	<0.001
	Type-D personality	0.41	0.11–0.72	0.008
	Interaction (Type-D × Inv)	0.22	0.05–0.39	0.012
	Age	0.02	−0.01–0.04	0.18
ODI	InvasivenessScore	6.20	5.10–7.30	<0.001
	Type-D personality	3.10	1.40–4.80	0.002
PSI	InvasivenessScore	−0.38	−0.47–−0.29	<0.001

**Table 4:** Multivariable logistic regression model for postoperative complications.

Predictor	OR	95% CI	*p*
InvasivenessScore	1.64	1.12–2.41	0.01
Type-D personality	1.32	0.78–2.11	0.21
Age	1.01	0.98–1.04	0.42
BMI	1.03	0.97–1.09	0.30

## Data Availability

The data presented in this study are available on reasonable request from the corresponding author. The data are not publicly available due to privacy and ethical restrictions.

## Supplementary Materials

The following supporting information can be downloaded at: https://www.mdpi.com/article/10.3390/jcm15093189/s1. Table S1 (Sensitivity Analyses of Surgical Trauma Gradient Components and Robustness Models), Table S2 (Model-Derived Predicted Values for Postoperative Pain Across the Surgical Trauma Gradient Stratified by Type-D Personality), Figure S1 (Sensitivity analyses supporting the robustness and interpretability of the Surgical Trauma Gradient framework).

## Abbreviations

ANOVA	Analysis of Variance
BMI	Body Mass Index
CI	Confidence Interval
DS14	Type-D Scale-14
HRV	Heart Rate Variability
LBP	Low Back Pain
MIS	Minimally Invasive Surgery
MIS-D	Minimally Invasive Decompression
MIS-F	Minimally Invasive Fusion
NA	Negative Affectivity
NYHA	New York Heart Association
ODI	Oswestry Disability Index
O-D	Open Decompression
O-F	Open Fusion
OR	Odds Ratio
PSI	Patient Satisfaction Index
SI	Social Inhibition
TLIF	Transforaminal Lumbar Interbody Fusion
tVNS	Transcutaneous Vagus Nerve Stimulation
VAS	Visual Analog Scale
