# Supplementary material for: Surgical Trauma Gradient as an Independent Predictor of Postoperative Pain, Functional Recovery, and Complication Risk After Spine Surgery: A 2 × 2 Invasiveness Model with Psychosocial Interaction
Source: J Clin Med. 2026 Apr 22;15(9):3189. doi: 10.3390/jcm15093189 (PMC13163500; doi:10.3390/jcm15093189)
Supplement: Supplementary file 1 [file jcm-15-03189-s001.zip › Supplementary Figure S1_Caption.pdf]

Figure S1. Sensitivity analyses supporting the robustness and interpretability of the Surgical Trauma Gradient framework.

(A) Forest plot illustrating the independent associations of exposure-related invasiveness (open vs. minimally invasive) and biomechanical strategy (fusion vs. decompression) with postoperative outcomes. Both dimensions demonstrate significant and independent contributions to pain intensity (VAS), functional disability (ODI), and patient satisfaction (PSI), supporting the conceptual validity of the 2×2 Surgical Trauma Gradient model.

(B) Sensitivity analyses demonstrating the stability of the association between surgical burden (InvasivenessScore) and postoperative pain across multiple model specifications, including exclusion of patients with prior spine surgery and stratification by pain duration. Effect estimates remain consistent in magnitude and direction, indicating robustness of the gradient effect.

(C) Model-derived predicted values for postoperative pain (VAS) across the Surgical Trauma Gradient stratified by Type-D personality. The increasing divergence between curves demonstrates a statistically significant interaction, indicating a clinically meaningful effect modification, whereby psychosocial vulnerability amplifies the impact of surgical burden on postoperative pain.
