# Supplementary material for: Surgical Trauma Gradient as an Independent Predictor of Postoperative Pain, Functional Recovery, and Complication Risk After Spine Surgery: A 2 × 2 Invasiveness Model with Psychosocial Interaction
Source: J Clin Med. 2026 Apr 22;15(9):3189. doi: 10.3390/jcm15093189 (PMC13163500; doi:10.3390/jcm15093189)
Supplement: Supplementary file 1 [file jcm-15-03189-s001.zip › Supplementary Table S1.pdf]

Supplementary Table S1. Sensitivity Analyses of Surgical Trauma Gradient Components and Robustness Models

Multivariable regression analyses evaluating the independent contributions of exposure-related invasiveness (minimally invasive vs. open) and biomechanical strategy (decompression vs. fusion), as well as sensitivity analyses excluding prior spine surgery and stratifying by pain duration. Models adjusted for age, sex, BMI, pain duration (where applicable), and Type-D personality.

A. Separate Modeling of Exposure (Minimally Invasive vs. Open)

Outcome	Predictor	β	95% CI	p-value
VAS (12 months)	Open vs. MIS	0.58	0.44 – 0.72	<0.001
ODI (12 months)	Open vs. MIS	5.10	4.00 – 6.20	<0.001
PSI (12 months)	Open vs. MIS	−0.32	−0.41 – −0.23	<0.001

B. Separate Modeling of Biomechanical Strategy (Fusion vs. Decompression)

Outcome	Predictor	β	95% CI	p-value
VAS (12 months)	Fusion vs. Decompression	0.64	0.50 – 0.78	<0.001
ODI (12 months)	Fusion vs. Decompression	6.45	5.30 – 7.60	<0.001
PSI (12 months)	Fusion vs. Decompression	−0.35	−0.44 – −0.26	<0.001

C. Exclusion of Patients with Prior Spine Surgery

Outcome	Predictor	β	95% CI	p-value
VAS (12 months)	InvasivenessScore	0.66	0.52 – 0.80	<0.001
ODI (12 months)	InvasivenessScore	6.05	4.90 – 7.20	<0.001
PSI (12 months)	InvasivenessScore	−0.36	−0.45 – −0.27	<0.001

D. Stratified Analysis by Pain Duration (Quartiles)

Outcome	Pain Duration Quartile	β (InvasivenessScore)	95% CI	p-value
VAS	Q1 (short duration)	0.61	0.40 – 0.82	<0.001
VAS	Q2	0.65	0.46 – 0.84	<0.001
VAS	Q3	0.70	0.50 – 0.90	<0.001

Outcome	Pain Duration Quartile	β (InvasivenessScore)	95% CI	p-value
VAS	Q4 (long duration)	0.74	0.53 – 0.95	<0.001

Interpretation

Across all sensitivity analyses, both exposure-related invasiveness and biomechanical strategy remained independently associated with worse postoperative outcomes. The magnitude and direction of effects were consistent with the main analyses. Exclusion of prior spine surgery cases did not materially alter results, and stratification by pain duration demonstrated a stable gradient effect across all quartiles. These findings support the robustness and conceptual validity of the Surgical Trauma Gradient framework.
