# Supplementary material for: Surgical Trauma Gradient as an Independent Predictor of Postoperative Pain, Functional Recovery, and Complication Risk After Spine Surgery: A 2 × 2 Invasiveness Model with Psychosocial Interaction
Source: J Clin Med. 2026 Apr 22;15(9):3189. doi: 10.3390/jcm15093189 (PMC13163500; doi:10.3390/jcm15093189)
Supplement: Supplementary file 1 [file jcm-15-03189-s001.zip › Supplementary Table S2.pdf]

Supplementary Table S2. Model-Derived Predicted Values for Postoperative Pain Across the Surgical Trauma Gradient Stratified by Type-D Personality

Predicted 12-month pain intensity (VAS) derived from multivariable regression models including InvasivenessScore, Type-D personality, and their interaction term, adjusted for age, sex, BMI, and pain duration.

A. Predicted VAS by InvasivenessScore and Type-D Status

InvasivenessScore	Surgical Group	Non-Type-D (Predicted VAS)	Type-D (Predicted VAS)	Absolute Difference
1	MIS-D	2.05	2.45	+0.40
2	O-D	2.58	3.12	+0.54
3	MIS-F	3.18	3.90	+0.72
4	O-F	4.02	4.95	+0.93

B. Gradient Effect (Slope Interpretation)

Predictor	β	Interpretation
InvasivenessScore	0.69	Each step increase in surgical burden increases VAS by ~0.69 points
Type-D personality	0.41	Independent increase in baseline pain level
Interaction (Type-D × Invasiveness)	0.22	Additional increase in slope per invasiveness level in Type-D patients

---

C. Clinical Interpretation

The difference in predicted pain between Type-D and non-Type-D patients increases progressively across the Surgical Trauma Gradient, from approximately 0.40 VAS points in minimally invasive decompression to 0.93 VAS points in open fusion.

This pattern confirms a clinically relevant **effect modification**, indicating that psychosocial vulnerability amplifies the impact of surgical burden on postoperative pain in a dose–response manner.
