# Supplementary figures and images for: Surgical Trauma Gradient as an Independent Predictor of Postoperative Pain, Functional Recovery, and Complication Risk After Spine Surgery: A 2 × 2 Invasiveness Model with Psychosocial Interaction
Source: J Clin Med. 2026 Apr 22;15(9):3189. doi: 10.3390/jcm15093189 (PMC13163500; doi:10.3390/jcm15093189)

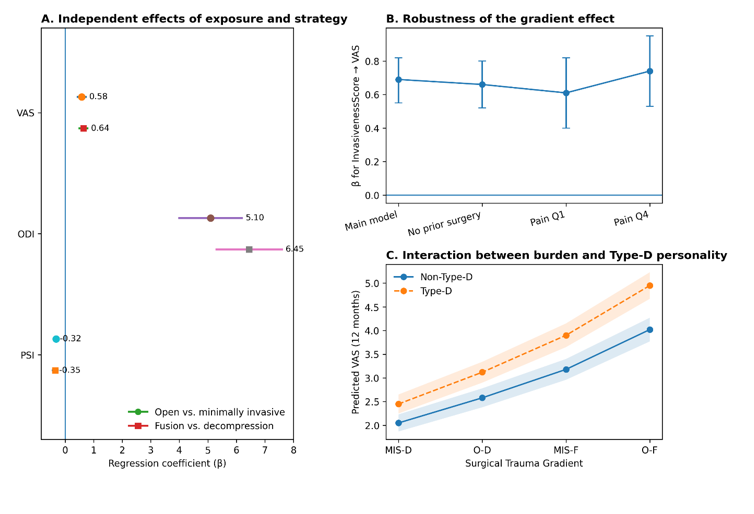

Supplement: Supplementary file 1 [file jcm-15-03189-s001.zip › Supplementary Figure S1.png]
